# Optimal Cutoffs for the Ratio of Arterial Oxygen Partial Pressure to Inspired Oxygen Fraction in Categorizing Respiratory Impairment Severity in Organ Failure Scores

**DOI:** 10.1111/aas.70137

**Published:** 2025-10-29

**Authors:** Anssi Pölkki, Matti Reinikainen, Bram Rochwerg, Christian Jung, Cornelius Sendagire, Dipayan Chaudhuri, Greg S. Martin, Tuomas Selander, Andrew Rhodes, Rui Moreno, Mervyn Singer, John G. Laffey, Pirkka T. Pekkarinen, the SOFA‐2 study group

**Affiliations:** ^1^ Department of Anaesthesiology and Intensive Care, Kuopio University Hospital and Institute of Clinical Medicine University of Eastern Finland Kuopio Finland; ^2^ Department of Medicine and Department of Health Research Methods, Evidence, and Impact McMaster University Hamilton Ontario Canada; ^3^ Department of Cardiology, Pulmonology and Vascular Medicine and Cardiovascular Research Institute Dusseldorf (CARID) Heinrich‐Heine‐University Duesseldorf Duesseldorf Germany; ^4^ Uganda Heart Institute Makerere University Kampala Uganda; ^5^ Division of Critical Care McMaster University and Department of Critical Care, St. Joseph's Healthcare Hamilton Ontario Canada; ^6^ Division of Pulmonary, Allergy, Critical Care and Sleep Medicine Emory University School of Medicine, and Grady Memorial Hospital Atlanta Georgia USA; ^7^ Science Service Center Kuopio University Hospital Kuopio Finland; ^8^ Adult Critical Care St. George's University Hospitals NHS Foundation Trust London UK; ^9^ Faculdade de Ciências Médicas de Lisboa, Nova Medical School, Lisboa, Portugal, Faculdade de Ciências da Saúde ULS de José, Lisboa, Portugal Covilhã Portugal; ^10^ Bloomsbury Institute of Intensive Care Medicine, Division of Medicine University College London London UK; ^11^ Anaesthesia and Intensive Care Medicine, School of Medicine University of Galway Galway Ireland; ^12^ Department of Anaesthesia Galway University Hospitals Galway Ireland; ^13^ Division of Intensive Care, Department of Anesthesiology and Intensive Care University of Helsinki and Helsinki University Hospital Helsinki Finland

## Abstract

**Background:**

The ratio of arterial oxygen partial pressure to fraction of inspired oxygen (PaO_2_/FiO_2_, hereafter P/F ratio) is a key component of the Sequential Organ Failure Assessment (SOFA) score. It reflects the severity of hypoxaemic respiratory failure. The ongoing revision of the SOFA score requires data‐driven cutoffs for P/F ratio as well as rational criteria for respiratory support. In this study, we aimed to determine the optimal P/F ratio cutoffs for determining respiratory failure categories in the revised SOFA score and examined whether advanced respiratory support should be a prerequisite for the most severe categories.

**Methods:**

We used the database of the intensive care unit of Kuopio University Hospital, Finland, for cutoff derivation and the eICU database, a multicenter U.S. intensive care registry, for external validation. We identified cutoffs most discriminative for hospital mortality using the log‐rank statistic test with the Contal and O'Quigley method. In external validation, these cutoffs were compared with those in the current respiratory SOFA score.

**Results:**

Optimal cutoffs were identified as follows: P/F ratio > 40 kPa (normal), 30–40 kPa (mild impairment), 20–30 kPa (moderate impairment), 10–20 kPa (severe impairment), and ≤ 10 kPa (critical impairment). These cutoffs resulted in clear separation of the severity categories (chi‐square for log‐rank statistic 356.9). They outperformed the current respiratory SOFA score cutoffs in the validation cohort (AUROC 0.615, 95% CI 0.607–0.622 vs. AUROC 0.610, 95% CI 0.603–0.618, *p* < 0.001). Advanced respiratory support was associated with higher mortality, but its inclusion as a prerequisite improved discrimination only in the moderately impaired respiratory function category, not in the severely or critically impaired categories.

**Conclusion:**

P/F ratio cutoffs using 10 kPa (75 mmHg) intervals were identified to be optimal for distinguishing stages of respiratory failure severity. The impact of respiratory support on P/F ratio–mortality associations suggests the need to calibrate any P/F ratio‐based score by support level, but optimal calibration methods require further study.

**Editorial Comment:**

In this study, the cut‐off values for the partial pressure of arterial oxygen to the fraction of inspired oxygen (P/F ratio) were investigated in a large Finnish intensive care database and validated externally with the US intensive care registry. The aim was to support a revision of the cut‐off values for the P/F ratio in the Sequential Organ Failure Assessment (SOFA) score. The results showed that incremental changes in the P/F ratio of 10 kPa are better than 13 kPa and emphasize the need for critical assessment of the current SOFA score.

## 1 Introduction

Respiratory failure is one of the major reasons for admission to the intensive care unit (ICU), either as an isolated impairment or as part of the multiorgan failure process. It is among the leading causes of mortality in ICU patients [1, 2]. A straightforward method to estimate the severity of respiratory failure is to measure the partial pressure of oxygen in arterial blood, which depends on the fraction of oxygen in inhaled gas, the lung gas exchange capacity, airway pressure (especially positive end‐expiratory pressure, driving pressure, PEEP), and ventilation [3, 4]. The ratio of arterial oxygen partial pressure to the fraction of oxygen in inhaled gas (PaO_2_/FiO_2_ ratio, hereafter P/F ratio) has become an established measure for the severity of hypoxaemic respiratory failure.

Due to its simplicity, P/F ratio is widely used in organ failure scores, such as the Sequential Organ Failure Assessment (SOFA) score [5], Multiple Organ Dysfunction Score (MODS) [6], and Logistic Organ Dysfunction Score (LODS) [7]. Along with the requirement for PEEP, P/F ratio also forms the basis of the Berlin criteria and its proposed 2024 update for acute respiratory distress syndrome (ARDS) definition [8, 9].

However, the P/F ratio cutoffs used to differentiate stages of respiratory failure are arbitrary and differ between the scoring systems. The cutoff intervals vary from 10 kPa (75 mmHg) in MODS [7] to 13.3 kPa (100 mmHg) in the ARDS criteria [8] and SOFA score [5], while LODS uses a single threshold of 20 kPa (150 mmHg) [7].

Moreover, it remains unclear how to best include mechanical ventilation and other modes of respiratory support into the severity definition. The P/F ratio may be dependent on the level of PEEP and other measurements of respiratory dynamics, suggesting the importance of calibration based on respiratory support.

The SOFA score, the most widely used organ failure score, is currently being updated after remaining unchanged for three decades [10]. Therefore, reassessing the P/F cutoffs for respiratory failure severity scoring is timely. Justifications for revising the respiratory SOFA score include: (1) mortality clearly increases only at a current respiratory SOFA score of 3 or higher, indicating poor discrimination at lower scores [11]; and (2) patients on room air (FiO_2_ 0.21) must have a PaO_2_ > 11.2 kPa (84 mmHg) to score 0. That high threshold may misclassify especially elderly patients without true respiratory dysfunction.

This study was conducted to support the SOFA 2 respiratory working group's decision‐making as part of the larger SOFA update committee. The primary objective was to establish practical data‐driven cutoffs for the P/F ratio that more consistently correlate with increasing mortality risk. We also aimed to determine whether the need for advanced respiratory support [non‐invasive ventilation (NIV) or invasive mechanical ventilation (IMV)] adds predictive value in addition to the P/F ratio. We planned to derive the optimal P/F cutoffs for determining severities of respiratory failure using a single‐center population with subsequent external validation using an extensive multicenter database.

## 2 Methods

We conducted a registry‐based study using a single‐center cohort in the derivation phase and a multicenter database design for external validation. Permission for derivation cohort data use was obtained from Kuopio University Hospital (KUH) administration in Finland (reference number: 478/2021). No interventions were performed on the study patients. Following Finnish legislation, the need to obtain individual patient consent was waived.

### 2.1 Study Population and Variables

For the derivation cohort, we included consecutive patients admitted to the mixed medical‐surgical ICU at KUH between January 1, 2013 and July 31, 2018. Patients for scheduled surgery, patients admitted solely for potential organ donation, and all readmissions were excluded. For cutoff validation, we used the eICU database, which includes admissions from different types of ICUs in the United States during 2014–2015 and is freely available to registered researchers [12]. We included adult patients aged over 18 years who were admitted to the ICU, with at least one P/F value recorded and data on respiratory support measured during the first 24 h, as well as data on vitality status at hospital discharge.

We used the P/F ratio data from the first 24 h after ICU admission and chose the lowest value recorded during that period. In patients with conventional oxygen therapy (COT) such as low‐flow or high‐flow nasal cannula (HFNC), we assumed an FiO_2_ of 0.3 in cases where the accurate FiO_2_ was not attainable. This assumption was made based on observations that the actual FiO_2_ at the trachea is at least around 0.3 with commonly used flow rates of 2–4 L/min in clinical intensive care practice [13].

For the derivation cohort, information describing the level of respiratory support was based on logged therapeutic intervention scoring system (TISS‐76) parameters or a separate record of advanced respiratory support [14]. According to the logic of the TISS‐76 system, if the patient received different levels of respiratory support during the first 24 h, then the most intensive level used was recorded.

### 2.2 Preset Requirements for the Cutoffs

According to the SOFA score framework, we aimed to determine five severity categories for impairment of respiratory function (described as normal, and mildly, moderately, severely, and critically impaired respiratory function). Preset requirements included that we did not require a linear increase in mortality across categories. Instead, we searched for cutoffs for the P/F ratio that would result in the clearest distinction between severity categories using the risk of death as a proxy. We decided a priori that if the statistical method would provide multiple sets of cutoffs with similar associations with the risk of death, we would prioritize cutoffs that are easy to remember and use in clinical practice.

### 2.3 Statistical Methods

We used death during the hospitalization as the primary outcome.

In the cutoff derivation phase, we first determined the distribution of the P/F ratio for each level of respiratory support: no respiratory support, COT (including use of HFNC), NIV, and IMV. The distribution was visualized using kernel density estimation. The use of HFNC was not recorded separately in the databases. Thus, we analyzed patients on HFNC along with others in the COT group.

We then determined the P/F ratio cutoffs for defining different levels of respiratory impairment using log‐rank survival analysis. The goal was to identify cutoffs that would provide the most significant separation in mortality between the five groups. Thus, we searched for the cutoffs resulting in the highest chi‐square log‐rank statistic, using the Contal and O'Quigley method [15]. We used P/F ratio values ranging from the 5th to the 95th percentile, in 1% increments, as candidate cutoffs. To identify the set of cutoffs yielding the highest log‐rank test statistic, all possible combinations of these candidate cutoffs were evaluated. Eventually, we rounded the cutoffs to the nearest practical and easily memorable value. We used Kaplan–Meier plots with pairwise comparisons between adjacent categories to depict separation using the log‐rank test (Mantel–Cox). We used the cutoffs from the original SOFA score (100 mmHg, i.e., 13.3 kPa intervals) as the reference for comparison.

Subsequently, we determined whether adding the receipt of advanced respiratory support (NIV or IMV) as an additional criterion for respiratory impairment levels improves the goodness of fit for the P/F cutoff‐based criteria. To do this, we assessed the impact of including the need for advanced respiratory support at the levels of mild to critical, moderate to critical, or severe to critical impairment stage. We performed this testing using the likelihood ratio test.

We assessed the predictive ability of the cutoffs using the Area Under the Receiver Operating Characteristic (AUROC). We compared the AUROC results to those obtained using the original respiratory SOFA score cutoffs using the DeLong test.

In the validation cohort, we performed further sensitivity analyses to determine whether the cutoffs function differently in specific subgroups. The subgroups included patients admitted to the ICU for respiratory‐related as compared to non‐respiratory‐related diagnoses as the admission cause, patients requiring advanced respiratory support during the first 24 h in the ICU as compared to those not requiring it, and patients in different age quartiles.

The differences between categorical variables were assessed using the chi‐square test.

We performed the statistical analyses using IBM SPSS Statistics, version 29, and R version 4.3.2 (packages: KMsurv, survival, survMisc, pROC).

## 3 Results

The derivation cohort consisted of 5848 patients. The majority of these (63%) were male, and the median age was 62 years. ICU mortality in the cohort was 8% and hospital mortality was 13%. The more detailed characteristics of study patients are described in Table 1.

**TABLE 1:** The background characteristics of patients in the derivation and validation cohorts.

Characteristic	Derivation cohort	Validation cohort
Number	5848	38,843
Sex (male)	3665 (62.7%)	21,793 (56.1%)
Age (median, IQR)	62 (51–70)	65 (54–75)
ICU mortality	464 (7.9%)	4953 (12.8%)
Hospital mortality	754 (12.9%)	7187 (18.5%)
Type of ICU admitted		
Medical‐surgical ICU	5848 (100%)	18,665 (48.1%)
Cardiac/cardiothoracic ICU	0	11,097 (28.5%)
Medical ICU	0	4109 (5.6%)
Surgical ICU	0	2780 (7.2%)
Neuro ICU	0	2192 (5.6%)
Level of respiratory support		
None	830 (14.2%)	1531 (3.9%)
Conventional oxygen therapy	1819 (31.1%)	7778 (20.0%)
Non‐invasive ventilation	543 (9.3%)	4301 (11.1%)
Invasive mechanical ventilation	2656 (45.4%)	25,233 (65.0%)
Median P/F ratio	32.6 kPa (24.2–42.6 kPa) 245 mmHg (182–320 mmHg)	29.6 kPa (19.5–42.4 kPa) 222 mmHg (146–318 mmHg)
Patient admitted from		
Emergency department	3291 (56.3%)	12,960 (33.4%)
Operation theater or post‐anesthesia care unit	1276 (21.8%)	7725 (19.9%)
Ward	751 (12.8%)	4538 (11.7%)
Another ICU	67 (1.1%)	36 (0.1%)
Another surveillance unit	160 (2.7%)	747 (1.9%)
Other/unknown	265 (4.5%)	12,838 (33.0%)
Leading cause of admission		
Cardiothoracic surgery	359 (6.1%)	7093 (18,2%)
Other cardiovascular cause	531 (9.0%)	4088 (10.5%)
Post cardiac arrest	197 (3.3%)	2600 (6.7%)
Sepsis	298 (5.0%)	5975 (15.3%)
Gastrointestinal	980 (9.9%)	2273 (7.0%)
Drug overdose	182 (3.1%)	1133 (2.9%)
Metabolic/renal	510 (8.7%)	1528 (3.6%)
Neurologic	1285 (21.9%)	3486 (8.9%)
Respiratory	631 (10.7%)	7550 (19.4%)
Trauma	648 (11.0%)	1101 (2.8%)
Other/unknown	306 (5.2%)	1338 (3.4%)

Abbreviations: ICU, intensive care unit, IQR, inter‐quartile range; P/F ratio, ratio of arterial oxygen partial pressure to inspired oxygen fraction.

**FIGURE 1:**
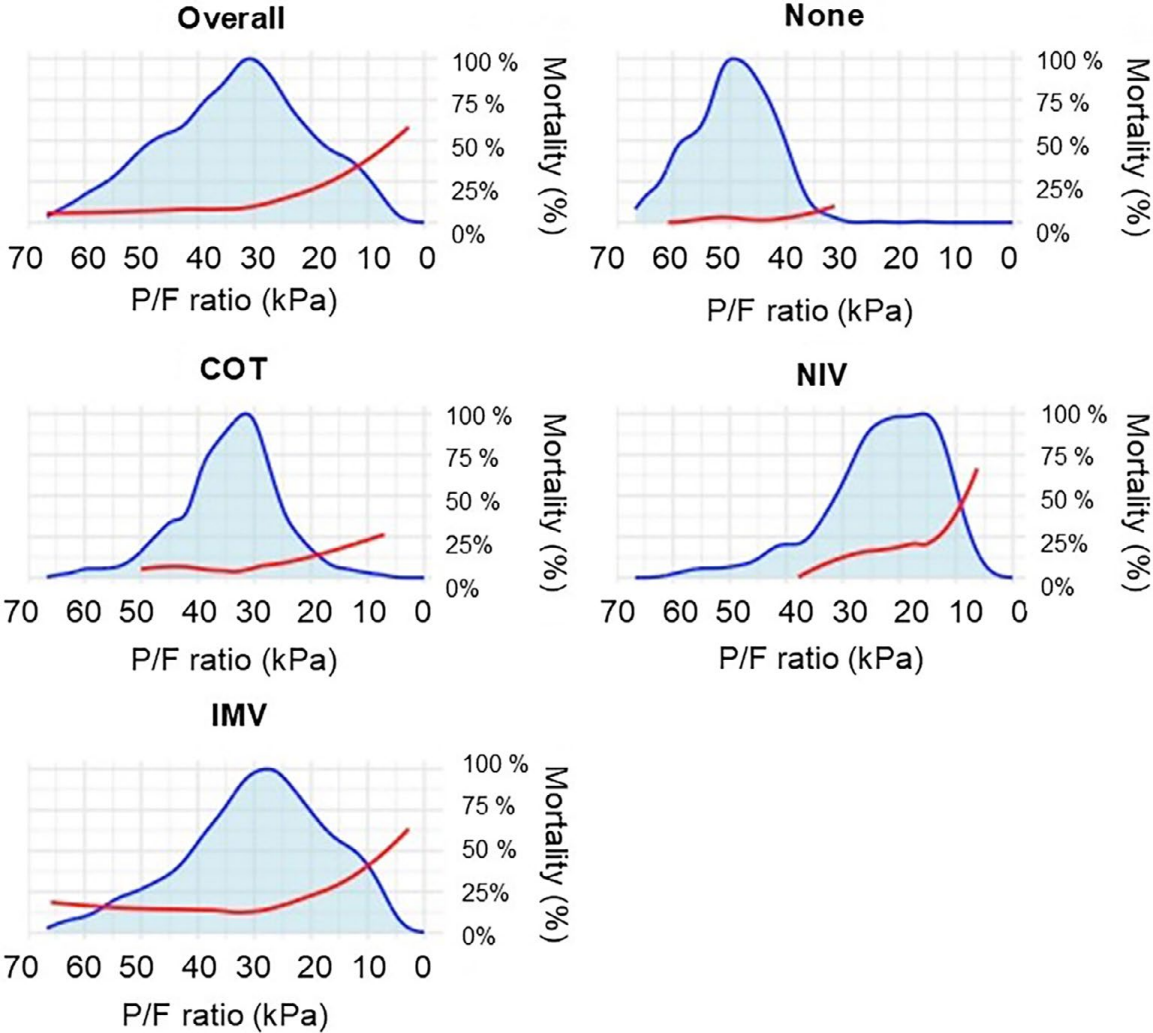
The distribution of patients by oxygen partial pressure to inspired oxygen fraction (P/F) ratio and the associated hospital mortality in the derivation cohort (*n* = 5848). The cohorts of patients breathing room air with no respiratory support (none), those treated with conventional oxygen therapy (COT), patients receiving non‐invasive ventilation (NIV), and those on invasive mechanical ventilation (IMV) are reported in separate panels, along with an overall population panel. The distribution, illustrated by the kernel density function, is shown as a grey area, while hospital mortality is represented by a red line with a locally estimated scatterplot smoothing (LOESS) curve.

The distribution of the P/F ratio varied across different levels of respiratory support. The median P/F ratio was highest in patients breathing room air, 49.5 kPa (Interquartile Range [IQR] 44.6–55.2 kPa). More intensive respiratory support was associated with lower P/F ratios: the median P/F ratio was 33.6 kPa (IQR 28.9–39.3 kPa) in patients treated with COT, and 20.9 kPa (IQR 15.3–28.0 kPa) in patients treated with NIV. Patients receiving IMV had a broader distribution of P/F ratio (median 28.6 kPa, IQR 20.9–39.0 kPa). The share of patients receiving advanced respiratory support with a P/F ratio below 20 kPa was 26.9%, and below 10 kPa 4.7%, while among patients not receiving advanced respiratory support, these percentages were 3.2% and 0.3%, respectively (Figure 1).

Hospital mortality was 2.4% in patients without any respiratory support, 6.5% in those receiving COT, 19.0% in those receiving NIV, and 19.3% in those treated with IMV (Figure 1). In the COT group, the exact FiO_2_ corresponding to the lowest P/F ratio was missing for 84.7% of patients. Of the patients receiving advanced respiratory support, 47.7% had a P/F ratio below 200, 26.6 kPa, and 11.1% had a ratio below 13 kPa. Among those not receiving advanced respiratory support, 21.7% had a P/F ratio below 26.6 kPa, and 3.1% had a ratio below 13.3 kPa.

### 3.1 Definition of P/F Cutoffs

According to the predetermined cutoff requirements, we identified four optimal P/F cutoff points to define five categories of respiratory failure severity. The two sets of cutoffs that resulted in the most distinct separation between groups (see Table e1 in the Supporting Information) included a set very close to 10 kPa intervals (9.8, 19.7, 29.6, and 39.6 kPa, log‐rank statistic 354.2) and another one with less practical intervals (11.2, 22.2, 33.4, and 44.5 kPa, log‐rank statistic 354.7). The third‐best set of cutoffs was close to the 13.3 kPa (100 mmHg) intervals used in the original SOFA score (13.2, 26.2, 39.6, 52.6 kPa, log‐rank statistic 343.6). As decided a priori, out of the two best sets of cutoffs with almost equal log‐rank statistic value, we gave priority to the more practical set. Therefore, we chose cutoffs of 10 kPa intervals with convenient rounding (Table 2). For rounded values, the chi‐square for log‐rank statistic pooled over strata was 356.9.

**TABLE 2:** The arterial oxygen partial pressure to inspired oxygen fraction (P/F) ratio cutoffs used and the descriptive definitions of the categories.

P/F ratio range	Respiratory function
P/F ratio > 40 kPa (300 mmHg)	Normal function
40 kPa (300 mmHg) ≥ P/F ratio > 30 kPa (225 mmHg)	Mild impairment
30 kPa (225 mmHg) ≥ P/F ratio > 20 kPa (150 mmHg)	Moderate impairment
20 kPa (150 mmHg) ≥ P/F ratio > 10 kPa (75 mmHg)	Severe impairment
10 kPa (75 mmHg) > P/F ratio	Critical impairment

**FIGURE 2:**
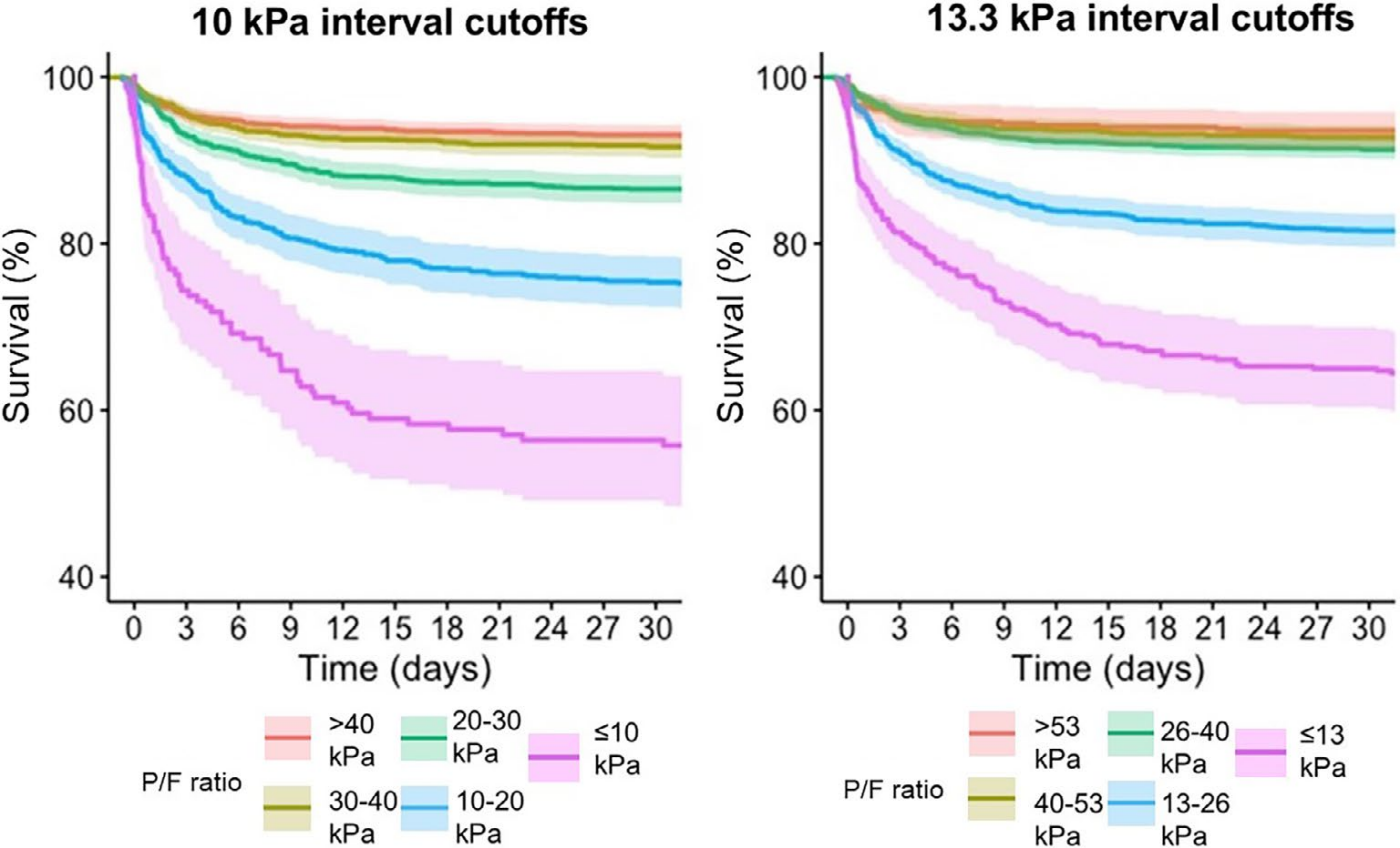
A Kaplan–Meier plot demonstrating the separation in survival between the severity categories of respiratory dysfunction, when categories were based on 10 kPa (75 mmHg) intervals between arterial oxygen partial pressure and inspired oxygen fraction (P/F) ratio cutoffs (left panel) or 13.3 kPa (100 mmHg) intervals between P/F ratio cutoffs (right panel).

The grouping cutoffs with 10 kPa intervals resulted in a more distinct separation between the groups compared to the original SOFA grouping of 13.3 kPa P/F ratio intervals (Figure 2). In the log‐rank test, the separation was statistically significant between all 10 kPa groups, whereas some of the original SOFA groups were overlapping (Table 3).

**TABLE 3:** The results of the log‐rank statistic test for pairwise comparisons of the adjacent categories in the derivation cohort: The chi‐square estimates and *p* values.

Pairwise comparisons of respiratory function categories: 10 kPa intervals between P/F ratio cutoffs	Chi square	Pairwise comparisons of respiratory function categories: 13.3 kPa intervals between P/F ratio cutoffs	Chi square
Normal vs. mildly impaired respiratory function (> 40 kPa vs. 30–40 kPa)	4.9 (*p* = 0.03)	> 53.3 kPa vs. 40–53.3 kPa	1.5 (*p* = 0.2)
Mildly vs. moderately impaired respiratory function (30–40 kPa vs. 20–30 kPa)	20.6 (*p* < 0.001)	40–53.3 kPa vs. 26.6–40 kPa	2.9 (*p* = 0.09)
Moderately vs. severely impaired respiratory function (20–30 kPa vs. 10–20 kPa)	44.7 (*p* < 0.001)	26.6–40 kPa vs. 13.3–26.6 kPa	84.9 (*p* < 0.001)
Critically vs. severely impaired respiratory function (10–20 kPa vs. ≤ 10 kPa)	23.8 (*p* < 0.001)	13.3–26.6 kPa vs. ≤ 13.3 kPa	41.3 (*p* < 0.001)

**FIGURE 3:**
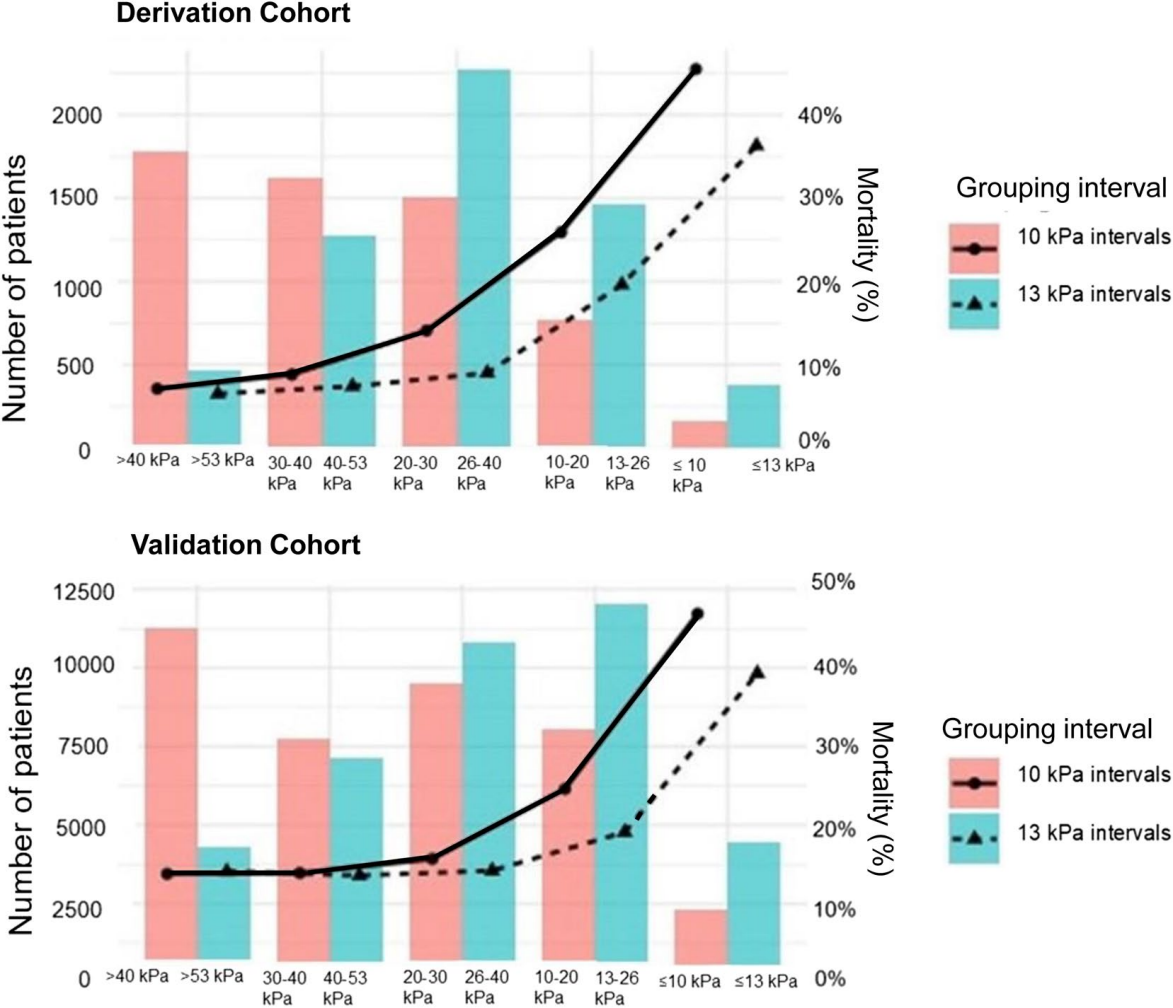
The number of patients (left *y*‐axis) categorized in each respiratory impairment category (*x*‐axis) is depicted with red bars for the 10 kPa (75 mmHg) grouping intervals and turquoise bars for the 13.3 kPa (100 mmHg) grouping intervals. Mortality (right *y*‐axis) in each category is shown with lines: The solid line with filled circles represents the 10 kPa grouping intervals, while the dashed line with triangles represents the current respiratory 13.3 kPa grouping intervals.

When a 10 kPa cutoff interval was used, patient numbers decreased and mortality increased with increasing severity category. With the 13.3 kPa cutoff interval, the 26.6–40 kPa category had the highest number of patients, and a clear increase in mortality was only observed in the two most severe categories (Figure 3).

### 3.2 Effect of Respiratory Support

On the admission day, 9.3% of patients were on NIV and 45.4% on IMV; that is, 54.7% of patients received advanced respiratory support (NIV or IMV). Patients receiving advanced respiratory support had higher mortality (19.3%) compared to other patients (5.0%) (*p* < 0.001). Mortality did not differ between patients receiving IMV (19.3%) or NIV (19.0%) (*p* = 0.85).

When incorporating the receipt of advanced respiratory support (NIV or IMV) as a prerequisite for scoring respiratory impairment severity levels, the goodness of fit of the cutoffs improved only when advanced respiratory support was required for at least moderate respiratory impairment levels (corresponding to a P/F ratio < 30 kPa, chi‐square 12.9, *p* < 0.001). However, adding advanced respiratory support as a prerequisite did not improve the goodness of fit for higher levels of respiratory impairment. The change was not significant for severe impairment (P/F ratio 10–20 kPa) (chi‐square 0.55, *p* = 0.45) or critical impairment (P/F ratio ≤ 10 kPa) (chi‐square 0.003, *p* = 0.95).

### 3.3 External Validation Cohort

The external validation cohort, extracted from the eICU database, included 38,843 admissions to 285 ICUs across 175 hospitals. The distribution of ICUs by unit type is shown in Table 1. A majority of these patients were male (56.1%), and their median age was 65 years. Most of the patients (65.0%) were supported with IMV and 11.1% with NIV. The ICU mortality in this cohort was 12.8%, while the hospital mortality was 18.5% (Table 1).

There was a significant separation in survival between all respiratory impairment categories when the 10 kPa intervals were used, except for the distinction between normal and mildly impaired respiratory function. When the 13.3 kPa intervals were used, there was significant separation between the moderately impaired and severely impaired groups, and between the severely impaired and critically impaired respiratory function groups (Table 4).

**TABLE 4:** The results of the log‐rank statistic test for pairwise comparisons of the adjacent categories in the validation cohort: The chi‐square estimates and *p* values.

Pairwise comparisons of respiratory function categories: 75 mmHg intervals between P/F ratio cutoffs	Chi square	Pairwise comparisons of respiratory function categories: 100 mmHg intervals between P/F ratio cutoffs	Chi square
Normal vs. mildly impaired respiratory function (> 40 kPa vs. 30–40 kPa)	0.14 (*p* = 0.90)	> 53.3 vs. 40–53.3 kPa	0.85 (*p* = 0.35)
Mildly vs. moderately impaired respiratory function (30–40 kPa vs. 20–30 kPa)	10.4 (*p* = 0.001)	40–53.3 kPa vs. 26.6–40 kPa	1.3 (*p* = 0.25)
Moderately vs. severely impaired respiratory function (20–30 kPa vs. 10–20 kPa)	224.5 (*p* < 0.001)	26.6–40 kPa vs. 13.3–26.6 kPa	97.5 (*p* < 0.001)
Very severely vs. severely impaired respiratory function (10–20 kPa vs. ≤ 10 kPa)	547.5 (*p* < 0.001)	13.3–26.6 kPa vs. ≤ 13.3 kPa	848.7 (*p* < 0.001)

Abbreviation: P/F ratio, ratio of arterial oxygen partial pressure to inspired oxygen fraction.

Mortality prediction slightly improved when 10 kPa interval cutoffs were used (AUROC 0.615, 95% CI 0.607–0.622) compared to 13.3 kPa interval cutoffs (AUROC 0.610, 95% CI 0.603–0.618), *p* < 0.001. The 10 kPa intervals outperformed the 13.3 kPa intervals in patients admitted to the ICU for non‐respiratory causes, patients requiring and not requiring advanced respiratory support, and in two age quartile groups. The 13.3 kPa cutoffs did not outperform the 10 kPa cutoffs in any of the sensitivity analysis groups (See Figures e1–e3 in the Supporting Information).

## 4 Discussion

In this registry‐based study, we established data‐based cutoffs for the P/F ratio to divide patients into five categories of respiratory function, ranging from normal to critically impaired. The study was initiated by the respiratory section of the SOFA 2 working group with the intention of using data‐driven decision support for defining the severity stages of respiratory failure. We aimed to develop a descriptive scale for the severity of respiratory dysfunction, not a predictive model. We searched for cutoffs leading to the maximum separation between the severity categories while simultaneously being practical for clinical use. Using cut‐offs ranging from 10 to 40 kPa with 10 kPa intervals (one of the two equal best sets of cutoffs provided by our data‐driven approach) resulted in good separation between the categories in hospital mortality. The cutoffs proved to be practical in a large external multicenter ICU population, and the AUROC‐based prediction ability was more accurate compared to the 13.3 kPa intervals used in the SOFA score and the ARDS Berlin criteria [8, 9, 16]. A further support for these cutoffs is that they can be easily converted into mmHg (10 kPa equals 75 mmHg within the accuracy of a standard blood gas analysis device). Of note is that the cutoffs generated by the data‐based approach in this study are identical to those used in the MODS score.

The advanced respiratory support (NIV or IMV) need was associated with a higher mortality risk, but this risk may stem from broader organ impairment rather than just respiratory failure. The hospital mortality of intubated patients on IMV plateaued at a level above 10% with P/F between 55 and 30 kPa, probably reflecting the impact of neurological and other conditions requiring airway protection in the absence of respiratory dysfunction. However, in patients supported with NIV, a lower P/F ratio appeared to be associated with higher hospital mortality throughout the range. Including the prerequisite of advanced respiratory support for the two most severe categories did not improve the separation of the groups in terms of mortality.

The SOFA score was introduced in 1996 and has since become the most widely used measure of multiorgan failure. Its purpose has expanded to use as an outcome measure in interventional trials [17] and as part of diagnostic criteria in the SEPSIS‐3 definition [18]. However, there has been growing recognition of its outdatedness and a demand for its revision to better align with today's ICU practices [11, 19]. The definitions of the different stages of organ failure, including respiratory failure, should ideally be based on population data rather than solely on expert opinions.

The primary requirements for measures in organ failure scores are that they should be as simple as possible, reflect the function of the target organ system accurately, and be readily available in clinical practice [16, 20]. In this regard, the P/F ratio is a well‐recognized and widely adopted variable, since measuring arterial gas samples is common practice in general ICU patients and especially in patients with respiratory dysfunction. Our results indicate that 10 kPa (75 mmHg) categories of P/F ratio describe the range of respiratory function in intensive care better than the 13.3 kPa (100 mmHg) categories used by the SOFA and ARDS scores.

Measuring peripheral oxygen saturation (SpO_2_/FiO_2_ ratio) has been proposed as a “surrogate” for the P/F ratio. Indeed, several studies have demonstrated a correlation between the two, and there have been suggestions to use SpO_2_/FiO_2_ as an alternative for assessing disease severity, particularly in settings where blood gas analyzers may be unavailable, such as in developing regions [21, 22, 23, 24, 25]. However, SpO_2_/FiO_2_ has numerous drawbacks. It is inaccurate in hypoxic and low perfusion states. Moreover, it is subject to anemia and movement artifacts [26].

The P/F ratio is well established for measuring the severity of respiratory impairment [27, 28]. Nonetheless, numerous studies have shown that the P/F ratio is influenced by various factors. It is well known that ventilator settings, especially the application of PEEP, modify the P/F ratio [3, 4]. An interesting suggestion is to add PEEP to the equation by calculating a PaO_2_/(FiO_2_*PEEP)‐ratio [29]. Recruitment maneuvers and prone positioning are frequently used methods for improving ventilation–perfusion matching and thereby oxygenation [4, 30, 31, 32]. Gilissen et al. demonstrated that the classification can also be more accurate if a PaO_2_ value, standardized according to Dalton's law based on hypercapnia and atmospheric pressure, is used [33]. Feiner and Weiskopf evaluated a number of P/F ratio‐affecting factors, including respiratory quotient and hemoglobin level [28]. We acknowledge the limitations and sources of bias related to the P/F ratio. However, when selecting measures for organ failure scores, it is essential to keep the scoring system as simple as possible to ensure its practical application.

## 5 Limitations

We acknowledge that the registry‐based study design introduces significant limitations. Although the cutoffs proved to be practical in a large external multicenter ICU population, applying multiple cutoffs simultaneously in the Contal and O'Quigley method may involve a risk of over‐optimization. Including only patients for whom the P/F ratio was available (for whom the arterial gas monitoring was deemed to be necessary) may have resulted in a selected patient cohort skewed toward the most severely ill individuals. We did not have data on clinical interventions that could affect P/F ratio values, such as prone positioning and recruitment maneuvers [4, 30]. Additionally, we did not have data on the non‐respiratory SOFA subscores, and therefore, we were unable to assess the magnitude of change in the respiratory SOFA score on the full SOFA score. We were unable to investigate the significance of trends in respiratory categories with different P/F ratio cutoffs (delta respiratory SOFA score) during the following ICU days, as we only had robustly validated data for the first 24 h of the ICU stay.

Data on the extracorporeal membrane oxygenation (ECMO) was also missing, but we believe the percentage of patients with ECMO was negligible in the cohorts. Moreover, for patients with ECMO at ICU admission, the worst P/F ratio was likely recorded before cannulation.

The use of HFNC has become justified even in moderate to severe respiratory failure [34, 35, 36]. Its use during this study's data collection was most probably low, but it is likely that the use has considerably increased since then [37]. It could be reasonable to categorize HFNC alongside advanced respiratory support techniques such as NIV and IMV. However, we were not reliably able to distinguish HFNC‐treated patients from other COT patients in this study.

In patients receiving COT, the effective FiO_2_ depends heavily on flow and the patient's minute ventilation [13]. For nearly 85% of the patients in the COT group, the exact FiO_2_ was unknown, and FiO2 of 0.3 was approximated. In practice, significant variation in effective FiO2 was likely among COT patients, and this approximation may have considerably affected the P/F ratio distribution.

## 6 Conclusion

P/F ratio categories based on cutoffs with 10 kPa (75 mmHg) intervals describe the range of respiratory function in intensive care better than the categories based on cutoffs with 13.3 kPa (100 mmHg) intervals used by the original SOFA score and ARDS definitions. The impact of the level of respiratory support on the association of P/F ratio with mortality supports the calibration of any P/F ratio‐based score according to the level of respiratory support. The method for optimal calibration requires further study.

## Author Contributions

A.P. and P.T.P. conceptualized and designed the study. P.T.P. developed the analytical methods and protocols in collaboration with A.P. A.P. conducted the formal data analysis with assistance from P.T.P., M.R., and T.S. A.P. and T.S. contributed to the software coding. A.P. prepared the tables and figures and drafted the manuscript. A.P., P.T.P., M.R., and J.G.L. provided substantial input on the initial interpretations of the results and made the first manuscript revisions. R.M., A.R., and M.S. make up the steering group of the SOFA‐2 revision committee, while A.P., P.T.P., B.R., C.J., C.S., D.C., and G.S.M. form the respiratory section under the supervision of J.G.L. All authors reviewed and contributed to the manuscript, approved the final version, and accepted full responsibility for its submission.

## Ethics Statement

The need to obtain ethics committee review was waived due to the retrospective nature of the study following the Finnish legislation.

## Conflicts of Interest

The authors declare no conflicts of interest.

## Supporting information


**Data S1:** aas70137‐sup‐0001‐Supinfo.pdf.

## Data Availability

The derivation cohort dataset was analyzed with permission from the Kuopio University Hospital (KUH) administration in Finland (reference number: 478/2021). This dataset is not publicly available. The validation cohort dataset was extracted from the eICU Collaborative Research Database, a large multi‐center critical care database made available by Philips Healthcare in partnership with the MIT Laboratory for Computational Physiology. Access to the eICU data can be requested at: https://eicu‐crd.mit.edu/.
