# Supplementary material for: Optimal Cutoffs for the Ratio of Arterial Oxygen Partial Pressure to Inspired Oxygen Fraction in Categorizing Respiratory Impairment Severity in Organ Failure Scores
Source: Acta Anaesthesiol Scand. 2025 Oct 29;70(1):e70137. doi: 10.1111/aas.70137 (PMC12571143; doi:10.1111/aas.70137)
Supplement: Supplementary file 1 — Data S1: aas70137‐sup‐0001‐Supinfo.pdf. [file AAS-70-0-s001.pdf]

Optimal cutoffs for ratio of arterial oxygen partial pressure to inspired oxygen fraction in categorizing respiratory impairment severity in organ failure scores

Online supplementary material

Content:

1. **Table of Contal and O´Quigley test results** Page 2
2. **Respiratory and non-respiratory admissions** Page 3
3. **Patients with and without advanced respiratory support:** Page 5
4. **Age groups (Age quartiles)** Page 6

Abbreviations:

LRS, Log Rank Statistic; P/F ratio, Arterial oxygen partial pressure to inspired oxygen fraction ratio; ICU, Intensive Care Unit; AUROC, Area Under the Receiver Operating Characteristic; SOFA score, Sequential Organ Failure Assessment score

eFigures:

eFigure 1 (Page 4): Mortality in each respiratory failure severity category in patients with and without respiratory related admissions cause for the intensive care

eFigure 2 (Page 5): Mortality in each respiratory failure severity category in patients requiring and not requiring advanced respiratory support (non-invasive or invasive mechanical ventilation).

eFigure 2 (Page 7): Mortality in each respiratory failure severitycategory in patients in four age groups.

1. Results of the Contal and O'Quigley test

We applied the Contal and O'Quigley test to identify four thresholds of the P/F ratio, aiming to find the cutoffs that provide the greatest separation between categories. The log-rank statistic (LRS) represents the degree of separation—the higher the LRS, the greater the separation between the groups.

c1	c2	c3	c4	LRS
26.7	53.3	80	106.7	254.11
25.7	51.5	75.9	102.9	254.13
25.3	50.7	76.9	101.3	260.88
26.1	52.4	78.5	104.9	263.03
24.8	49.7	74.5	99.5	264.95
21.3	42.8	64.3	85.6	275.05
23.7	47.5	71.2	94.9	281.11
24.3	48.7	72.9	97.3	281.44
22.0	44.0	66.0	88.0	282.55
20.7	41.5	62.1	82.9	284.84
22.9	46.0	69.1	92.1	286.79
20.0	40.0	60.0	53.3	290.40
17.7	35.5	39.9	70.9	293.40
22.5	51.7	67.6	90.1	294.09
19.3	38.7	58.0	77.3	299.66
16.8	36.6	50.4	67.3	304.36
18.4	36.9	55.5	74.0	305.97
16.0	32.0	48.0	64.1	310.62
14.9	30.0	45.1	55.3	316.53
12.1	24.3	36.4	48.7	333.76
14.0	29.2	42.0	56.0	334.31
13.1	26.3	39.7	52.5	343.59
9.9	19.7	29.6	39.5	354.21
11.1	22.3	33.3	44.5	354.73

Table e1. Columns c1, c2, c3, and c4 represent the tested cut-offs (in kPa). The rightmost panel represents the corresponding 24 highest log-rank statistic (LRS) with the corresponding cutoff combinations.

2. **Respiratory and non-respiratory admissions**

We conducted a subanalysis to assess whether the P/F ratio cutoff intervals (10 kPa vs. 13.3 kPa) perform consistently in patients admitted to the ICU for either respiratory or non-respiratory primary causes.

List of admission causes considered respiratory-related:

• Apnea-sleep; surgery for (i.e., UPPP - uvulopalatopharyngoplasty)

• Apnea, sleep • ARDS-adult respiratory distress syndrome, non-cardiogenic pulmonary edema

• Arrest, respiratory (without cardiac arrest)

• Asthma

• Atelectasis

• Biopsy, open lung

• Effusions (pleural)

• Emphysema/bronchitis

• Lung transplant

• Aspiration pneumonia

• Bacterial pneumonia

• Fungal pneumonia

• Pneumonia, other

• Parasitic pneumonia (i.e., Pneumocystic pneumonia)

• Viral pneumonia

• Pneumothorax

• Other medical respiratory disease

• Respiratory surgery for other reasons

• Thoracotomy for benign tumor (i.e. mediastinal chest wall mass, thymectomy)

• Thoracotomy for bronchopleural fistula

• Thoracotomy for lung cancer

• Thoracotomy for lung reduction

• Thoracotomy for other malignancy in chest

• Thoracotomy for other reasons

• Thoracotomy for pleural disease

• Bullectomy

• Pulmonary hemorrhage/hemoptysis

• Hemothorax

• Poisoning (carbon monoxide, arsenic, cyanide)

• Obstruction-airway (i.e., acute epiglottitis, post-extubation edema, foreign body, etc)

• Weaning from mechanical ventilation (transfer from other unit or hospital only)

In the validation cohort, the 10 kPa cutoff intervals improved discrimination in patients without respiratory failure as the primary ICU admission reason but offered no advantage in those admitted for respiratory failure. In patients without respiratory related cause as the main ICU admission reason, AUROC was 0.612 (95% CI: 0.603–0.620) for 10 kPa intervals and 0.608 (95% CI: 0.599–0.616) for 13.3 kPa intervals, with a significant difference ($p < 0.004$, DeLong test). In patients with respiratory failure as the main reason, AUROC was 0.616 (95% CI: 0.602–0.630) and 0.612 (95% CI: 0.599–0.612), respectively, with no significant difference ($p = 0.18$).

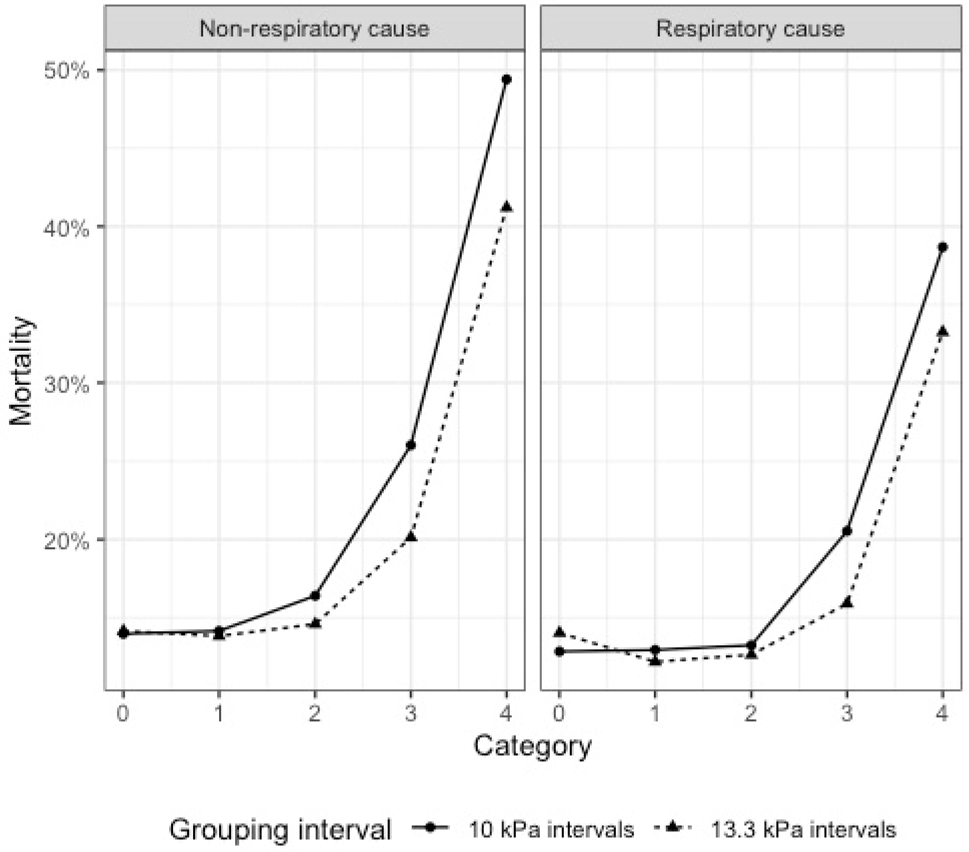

Figure e1. The left panel shows patients admitted for non-respiratory causes; the right panel, for respiratory causes (see respiratory disease related admission diagnosis list). Mortality trends (y-axis) by respiratory failure category: 0 = none, 1 = mild, 2 = moderate, 3 = severe, and 4 = critical. The solid line with circles shows mortality using 10 kPa cutoff intervals; the dashed line with triangles shows the current respiratory SOFA score (13.3 kPa intervals).

3. **Patients with and without advanced respiratory support:**

The 10 kPa cutoff intervals showed better discrimination for hospital mortality than the 13.3 kPa intervals in both patients with and without advanced respiratory support during the first 24 hours of the ICU stay. In patients receiving advanced respiratory support (n=29,534), the AUROC for predicting hospital mortality was 0.604 (95% CI: 0.5954–0.6121) using the 10 kPa cutoff, and 0.6001 (95% CI: 0.5918– 0.6084) using the 13.3 kPa cutoff. When compared using the DeLong test, the p-value was <0.001. In patients who did not require advanced respiratory support (n=9,309) during the first 24 hours, the AUROC for predicting hospital mortality was 0.650 (95% CI: 0.6335–0.667) with the 10 kPa cutoff, and 0.644 (95% CI: 0.6274–0.6606) with the 13.3 kPa cutoff. The DeLong test p-value was 0.02. Advanced respiratory support was considered as requiring either non-invasive or invasive mechanical ventilation.

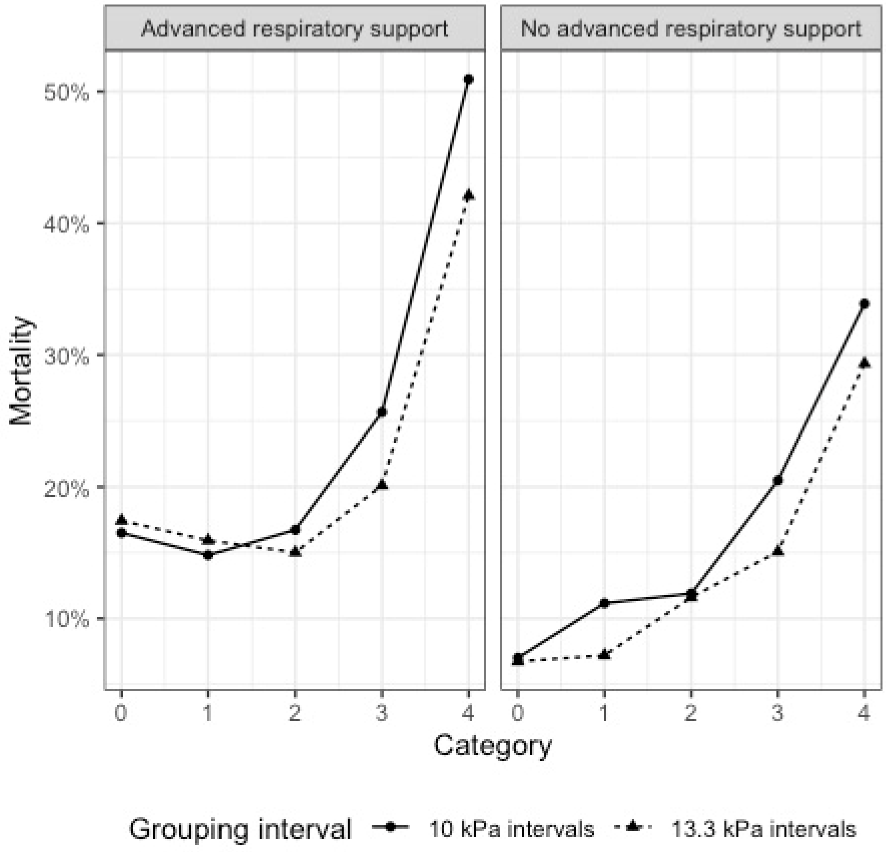

Figure e2. Mortality trends of patients categorised by different stages of respiratory failure: category 0 represents no respiratory failure; category 1, mild; category 2, moderate; category 3, severe; and category 4, critical respiratory failure. The solid line with filled circles shows mortality rates categorised using 10 kPa cutoff intervals, while the reference line (dashed line with triangles) represents the current respiratory Sequential Organ Failure Assessment (SOFA) score using 13.3 kPa cutoff intervals. The left panel depicts a subanalysis of patients who required advanced respiratory support within the first 24 hours after ICU admission, while the right panel depicts patients who did not require advanced respiratory support.

4. **Age groups (Age quartiles)**

To perform a sensitivity analysis on how the cutoff intervals perform across different age groups, we categorized patients into four age quartiles and evaluated the AUROC of P/F ratio cutoff intervals within each quartile. We compared the discriminatory ability (AUROC) using the DeLong test. The p-value indicates whether there is a statistically significant difference in discrimination between the two cutoff intervals, as measured by the DeLong test.

Age group 1 (0-54 years):

Number of cases: 9,751

PF ratio with 10 kPa intervals: AUROC = 0.638 (95% CI: 0.621–0.654)

PF ratio with 13.3 kPa intervals: AUROC = 0.640 (95% CI: 0.624–0.657), p = 0.33

Age group 2 (55-64 years):

Number of cases: 9,611

PF ratio with 10 kPa intervals: AUROC = 0.617 (95% CI: 0.601–0.633)

PF ratio with 13.3 kPa intervals: AUROC = 0.608 (95% CI: 0.592–0.624), p < 0.001

Age group 3 (65-74 years):

Number of cases: 9,715

PF ratio with 10 kPa intervals: AUROC = 0.602 (95% CI: 0.587–0.617)

PF ratio with 13.3 kPa intervals: AUROC = 0.599 (95% CI: 0.584–0.614), p = 0.21

Age group 4 (75 years and older):

Number of cases: 8,796

PF ratio with 10 kPa intervals: AUROC = 0.604 (95% CI: 0.590–0.618)

PF ratio with 13.3 kPa intervals: AUROC = 0.595 (95% CI: 0.581–0.610), p < 0.001.

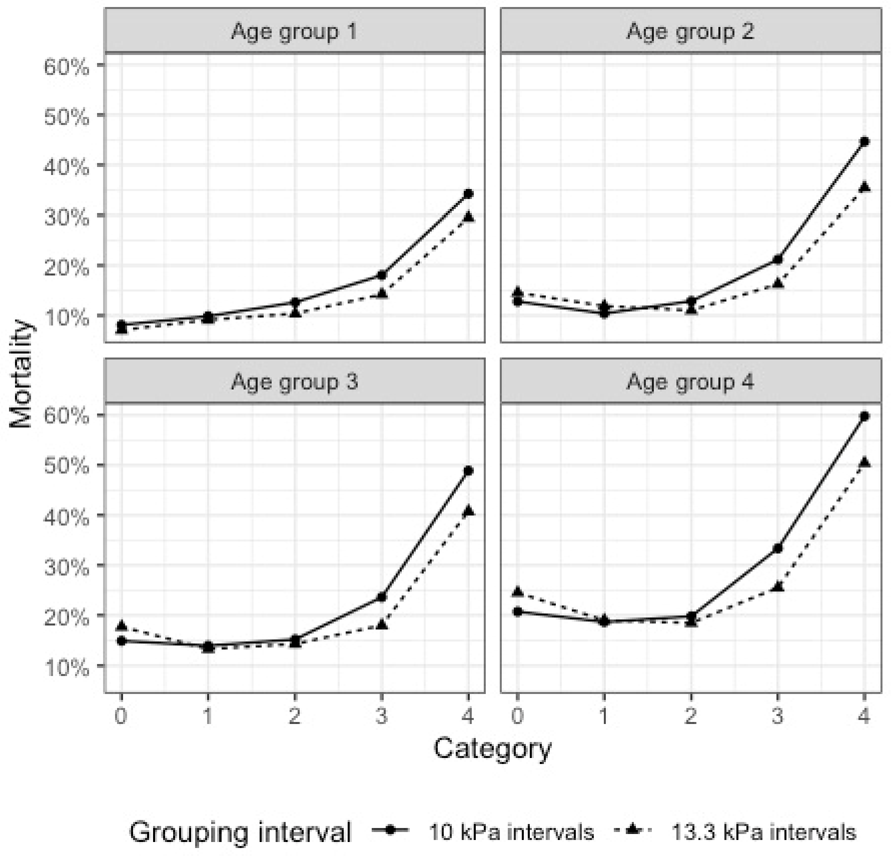

Figure e3. Patients admitted to the ICU across different age quartiles: age group 1 (0–54 years), age group 2 (55–64 years), age group 3 (65–74 years), and age group 4 (75 years and older). The x-axis in each age group panel shows patients categorized by stage of respiratory failure: category 0 = no respiratory failure; category 1 = mild; category 2 = moderate; category 3 = severe; and category 4 = critical respiratory failure. The solid line with filled circles shows mortality rates categorised using 10 kPa cutoff intervals, while the reference line (dashed line with triangles) represents the current respiratory SOFA score using 13.3 kPa cutoff intervals.
